# Effect of Roasting Levels and Drying Process of *Coffea canephora* on the Quality of Bioactive Compounds and Cytotoxicity

**DOI:** 10.3390/ijms19113407

**Published:** 2018-10-31

**Authors:** Deborah Bauer, Joel Abreu, Nathállia Jordão, Jeane Santos da Rosa, Otniel Freitas-Silva, Anderson Teodoro

**Affiliations:** 1 Laboratory of Functional Foods—Universidade Federal do Estado do Rio de Janeiro, Rio de Janeiro 22290-240, Brazil; bauer.deborah@hotmail.com (D.B.); pimenabreu@gmail.com (J.A.); 2 Food Science Departament, Universidade Federal do Estado do Rio de Janeiro, Rio de Janeiro 22290-240, Brazil; nathalliasomerhaldenathalliasomerhalder@gmail.com or allianath94@gmail.com; 3 Empresa Brasileira de Pesquisa Agropecuária—Embrapa Agroindústria de Alimentos, Rio de Janeiro 23020-470, Brazil; jeane@ctaa.embrapa.br or jeane.rosa@embrapa.br (J.S.d.R.); otniel.freitas@embrapa.br (O.F.-S.)

**Keywords:** green coffee, chlorogenic acids, roast coffee, Robusta, cancer

## Abstract

Coffee is a popular drink consumed all over the world. Besides its long-recognized stimulant effect, it has important nutritional and health effects. However, the type of bean processing modifies the composition of brewed coffee and possibly its bioactivity. In this study, extracts obtained from green and roasted beans of *Coffea canephora* (*Coffea canephora* var. robusta) were submitted to spray- or freeze-drying and were tested for antiproliferative activity, using MTT assay, and their influence on the cell cycle and apoptosis by flow cytometry analysis. Moreover, colors and nutrient contents were measured to identify the changes due to the roasting process. The results obtained showed that extracts from green and light roasted beans exhibited strong bioactive capacity. Coffee extracts promoted a decrease in cell viability, modulated cell cycle and induced apoptosis in human prostate carcinoma cell line (DU-145). The level of roasting reduced this property, but the type of drying did not in all cases.

## 1. Introduction

Coffee is one the most important food commodities in the world. About 60 tropical and subtropical countries produce coffee extensively, and in some cases, it is the main agricultural export product [1]. Brewed coffee is a major contributor to the dietary intake of polyphenols [2]. Besides the long-known stimulant effect, recent research has demonstrated the functional and protective potential of this beverage [3]. Raw coffee beans are rich in bioactive compounds, such as chlorogenic acids, trigonelline (hypoglycemic effects), caffeine, tocopherols and diterpenes.

Caffeine has different biological activities, such as stimulation of the central nervous system, myocardial stimulation, and peripheral vasoconstriction [4]. Phenols are a class of plant compounds with the potential to eliminate free radicals because of their stable structure after free-radical capture as hydroxycinnamic acids, with chlorogenic acids (CGA) and caffeic acid (CA) being the most abundant in coffee [5]. These compounds, found mainly in green coffee, are important and biologically active dietary polyphenols. CGA plays several important therapeutic roles, such as antioxidant, anti-inflammatory, antibacterial, antipyretic, hepato- and neuro-protective activities, and can help prevent retinal degeneration, obesity and hypertension. Likewise, it has been found that CGA can modulate lipid metabolism and glucose in both healthy people and those suffering from genetically related metabolic disorders. It is speculated that CGA performs crucial roles in lipid and glucose metabolism regulation and thus helps to treat many disorders, such as cardiovascular disease, diabetes and obesity. It is a free radical scavenger, and a central nervous system stimulator, and has DNA-protective and anticancer functions [6,7,8].

The profiles of these compounds are influenced mainly by genetic aspects, such as species and varieties, and physiological aspects, such as the degree of maturation [9,10]. Differences among species include physical aspects, chemical composition and beverage characteristics. The Robusta (*Coffea canephora* or *C. robusta*) variety is used for the production of standard-quality coffees. It produces a drink with pronounced bitterness and has high bioactive compound levels. It is more tolerant of growing heights and climates and is frequently used for instant coffee because of its low cost and high efficiency [11,12].

Roasting is the primary processing that coffee beans undergo. During roasting, the beans develop important flavor characteristics. This process affects the composition of the coffee beans and beverage, also influencing potential food bioactivity due to interactions occurring in the compounds available in the coffee matrix [13].

Epidemiologic data suggest that the high and regular consumption of some foods and beverages reduces the risk of chronic pathologies, such as cancer [14]. Prostate cancer (PCa) is the most frequent in men diagnosed with cancer in Brazil [15], and there is evidence that the acids contained in the coffee act to inhibit cancer [16,17]. The aim of this study was to evaluate and compare the bioactive compounds and antioxidant activity of Robusta coffee bean extracts obtained by spray- and freeze-drying after different roasting processes and their cytotoxic effects on a human prostate carcinoma cell line.

## 2. Results and Discussion

### 2.1. Physicochemical Results

#### 2.1.1. Colorimetry

As expected, the colors of the extracts were different according to the degree of roasting and also with the type of drying. In the CIE L* a* b* color diagrams, the parameter L* corresponds to the luminosity, so the values can range from 0 (black) to 100 (white); a* would be the chromatic coordinate of red (+) to green (−) and b*, chromatic coordinate from yellow (+) to blue (−). As seen in Table 1, the green extracts had higher L* values, with the spray-dried green extract (GS) being the one with lighter color, while the freeze-dried roasted extracts had the lowest luminosity. The values of the parameters a* and b* indicated shade from yellow to red, but still much to the center of the chromaticity diagram (gray region), indicating high saturation (low purity of color in relation to gray probably due the roasting process, since it is not totally homogeneous [18].

Bicho et al. [19] also concluded that the parameters L* had a consistent pattern of variation along the roasting, being a reliable measure to study the color change during coffee roasting.

The color modification of the coffee extract is due to complex biochemical mechanisms during roasting, such as Maillard reaction, Strecker degradation, caramelization of sugars, and degradation of chlorogenic acids, proteins and polysaccharides, which are responsible for the aroma and flavor of roasted coffee [9,20].

#### 2.1.2. Bioactive Properties of Aqueous Coffee Extract

Sucrose is the main carbohydrate in green coffee. Fructose is one of the main monosaccharides found in coffee beans and its content can vary as a consequence of postharvest processing [21,22]. These sugars were found only in samples of green coffee (Table 2). Sucrose is rapidly destroyed at the initial stage of roasting and is the main source of the aliphatic acids (formic, acetic, glycolic and lactic acids) produced during this process [23]. Therefore, it is not possible to detect sugars in the roasted coffee extracts.

Regarding the amino acids found in aqueous extracts of Robusta coffee, overall, the amino acid levels dropped by more than half in the roasted samples compared to the green samples (Table 2). Glutamine was the most abundant amino acid, followed by asparagine, without significant difference between the types of drying. In general, the concentration of amino acids was higher in Robusta than in Arabica variety. Carbohydrates and amino acids are the main components that contribute to the formation of the typical aroma during roasting. Asparagine and histidine are the main substrates to form acrylamide and 4-methyl imidazole in the roast process, respectively. These substances are potential carcinogens produced by the Maillard reaction [24]. The complete table with the levels of all the amino acids showing their composition and roles were reported by Murkovic and Derler [25].

**Figure 1:**
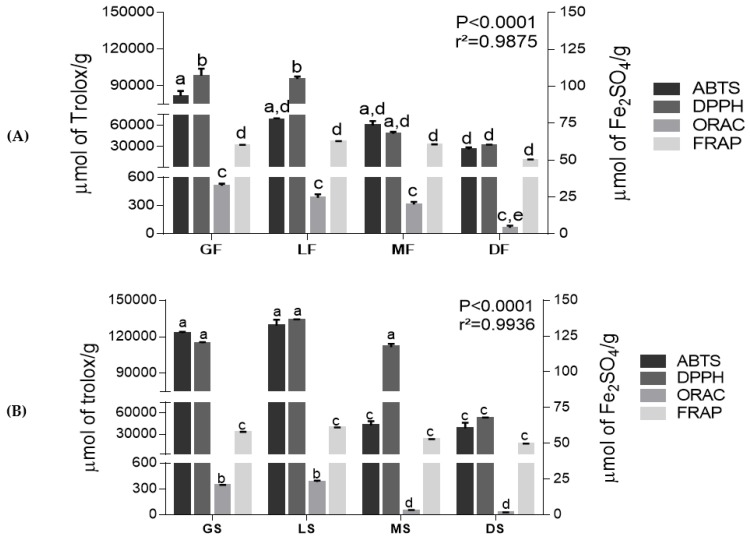
Antioxidant activity potential according to DPPH assay, Trolox Equivalent Antioxidant Capacity (ABTS/TEAC), Ferric Reducing Ability (FRAP) and Oxygen radical absorbance capacity (ORAC) analyses of freeze-dried (**A**) and spray-dried (**B**) coffee extracts. Different letters in the same analysis mean that samples are different (*p* < 0.05) and same letters indicate the samples are the same (*p* > 0.05). Abbreviations: GF—green freeze-dried, LF—light roasted freeze-dried, MF—medium roasted freeze-dried, DF—dark roasted freeze-dried, GS—green spray-dried, LS—light roasted spray-dried, MS—medium roasted spray-dried, DS—dark roasted spray-dried.

In the assays of antioxidant activity, the extracts were analyzed at concentrations of 5, 10 and 12.5 mg/L by DPPH assay, Trolox Equivalent Antioxidant Capacity (ABTS/TEAC), Ferric Reducing Ability (FRAP) and Oxygen radical absorbance capacity (ORAC) (Figure 1A,B). The green and light roasted samples presented a similar pattern in the antioxidant analyses. Green and light extracts of freeze-dried Robusta coffee had higher antioxidant potentials in this drying process. In spray-dried extracts, green spray-dried (GS) and light roasted spray-dried (LS) showed the highest potential in comparison with all extracts. The antioxidant capacity observed in roasted coffee extract could be due to the presence of melanoidins, end products of the Maillard reaction [26,27,28]. 

In ORAC analysis, lower antioxidant activity was observed, with the lowest values observed in spray-dried samples with a minimum value of 20.9 ± 6.7 µM Trolox eq./g and a maximum value in freeze-dried extracts of 510.8 ± 36.9 µM Trolox eq./g. Meanwhile, FRAP results did not show relevant changes in roast types and even in drying processes.

This can occur due to the fact that the ORAC method is performed with the peroxyl radical, which simulates a biological free radical. Some methods, such as FRAP, are based on Fe (III) complex that is a long-living free radical. As expected, these methods can present drawbacks because this synthetic free radical could not be compatible with food matrix natural antioxidants [29]. Studies have shown that no single test can estimate accurately the antioxidant activity in a sample, mainly because standardized methods should evaluate its availability against various oxygen reactive species and/or nitrogen reactive species, such as superoxide anion, hydroxyl (OH) and peroxynitrite, so this requires specific methods for specific radical sources. Thus, the use of different methods is necessary in antioxidant activity measurement and together these techniques could draw a reliable profile of the antioxidant content in a food matrix [30,31].

Changes in the antioxidative capacity of coffee upon roasting are also associated with the degradation of chlorogenic acid, but not necessarily, a reduction in antioxidant activity occurs. Chlorogenic acid fate in roasting can lead to other characteristic roasted products, such as chlorogenic acid lactones, and also minor cinnamic acid derivatives with antioxidant activity as well [27,32,33,34].

The caffeine content was affected by roasting (Table 3). However, the different drying processes did not influence the caffeine content of the extracts. The contents of this alkaloid in the beverage are influenced by the type and also by the process used in its preparation. During roasting, a small amount of caffeine is lost. Robusta coffee has a higher amount of caffeine than the Arabica’s one [35]. Chlorogenic acids and caffeine are found in brewed coffee because they are readily solubilized in hot water. Probably due to the existence of a variation of the content of the various compounds present in this beverage, a strong influence is observed by the processes used in its production chain and preparation for consumption [36].

The total phenolic compounds in the light roasted freeze-dried (LF), green freeze-dried (GF), green freeze-dried (GS) and light roasted spray-dried (LS) samples were higher, with values of 3792.0 ± 13.4, 3051.1 ± 33.7, 2816.6 ± 19.2 and 3046.4 ± 64.0 mg gallic acid equivalents/100 g, respectively. There was no significant difference between the GF, GS and LS (*p* > 0.05). The total content of phenolic compounds was modified in the extracts from roasted beans, and this content was lower in spray-dried samples, where a temperature close to 180 °C was used (Table 3).

Perrone [37] observed the influence of coffee roasting on incorporation of phenolic compounds in melanoidins and their relationship with antioxidant activity. They found values in Arabica coffee from 67.6 mg to 370.3 mg gallic acid equivalents/100 g, varying according to roasting time and cultivar analyzed. The values were lower compared to these in the present study. The composition of extract depends on the solvent used, the quality and the origin of the plant material, storage conditions and pretreatment [38,39].

The contribution of different coffee components to antioxidant activity of the brew is a topic of great interest in literature. Chlorogenic acids are considered to be the major contributors to the antioxidant activity of brewed coffee, followed by melanoidins, which are end products of the Maillard reaction [37,40]. The total chlorogenic acids content of green coffee beans can vary according to species and cultivars, the degree of maturation, agricultural practices, climate and soil [41,42].

The polyphenols undergo chemical modification or degradation upon roasting, and some products of their decomposition, like phenylindans, have high antioxidative activity [32,43]. In addition to the chlorogenic acids, other minor compounds of the chlorogenic acid family have been reported. Trace amounts of diferuloylquinic acids, dimethoxycinamoylquinic acids, caffeoyl-dimethoxycinamoylquinic acids and feruloyl-dimethoxycinamoylquinic acids were identified in Robusta coffee [42,44].

**Figure 2:**
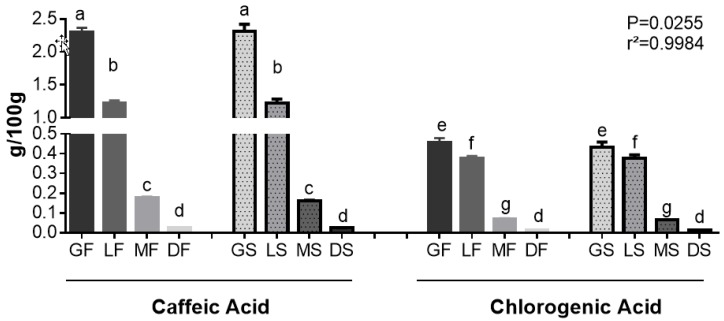
Caffeic and chlorogenic acids contents in the soluble extracts of green, light roasted, medium roasted and dark roasted Robusta coffee, submitted to freeze-drying and spray-drying. Different letters in the same analysis mean that samples are different (*p* < 0.05) and same letters indicate the samples are the same (*p* > 0.05). Abbreviations: GF—green freeze-dried, LF—light roasted freeze-dried, MF—medium roasted freeze-dried, DF—dark roasted freeze-dried, GS—green spray-dried, LS—light roasted spray-dried, MS—medium roasted spray-dried, DS—dark roasted spray-dried.

The data show that caffeic acid was the most abundant acid in the dry extracts of Robusta coffee (Figure 2). It is also possible to observe that the roasting process decreased the content of these compounds, but the drying process did not have an influence. The levels of bioactive compounds were lower in the medium and dark roasted samples. The caffeic acid content has a relationship with oxidative properties under thermal conditions. The first steps in the degradation of caffeic acid are related to the formation of decomposition products, which have lower antioxidant capacity when compared to the original molecule [45].

### 2.2. Cell Results

**Figure 3:**
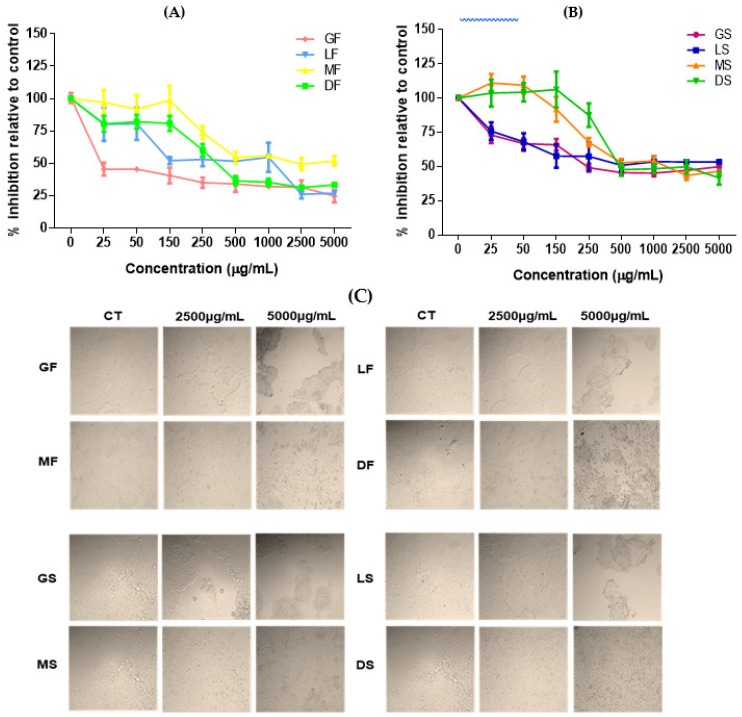
Percentage inhibition in comparison with control of viability of prostate tumor cells (DU-145) by the MTT method, after 24 h of treatment with freeze-dried (**A**) and spray-dried (**B**) aqueous coffee extracts at concentrations from 25 μg/mL to 5000 μg/mL. Photos of wells with concentrations of control, 2500 and 5000 μg/mL (**C**). Abbreviations: GF—green freeze-dried, LF—light roasted freeze-dried, MF—medium roasted freeze-dried, DF—dark roasted freeze-dried, GS—green spray-dried, LS—light roasted spray-dried, MS—medium roasted spray-dried, DS—dark roasted spray-dried.

We observed a significant increase in the inhibition of proliferation with low doses (25 and 50 μg/mL) of the green freeze-dried extract compared to other extracts (*p* < 0.05). The screening of anti-proliferative activity in PCa cells demonstrated that freeze-dried extracts were able to reduce cell viability significantly. However, the medium roasted freeze-dried (MF) extract presented the lowest inhibition (%) of viability (*p* < 0.05). The MTT (3-(4,5-dimethylthiazol-2-yl)-2,5-diphenyltetrazolium bromide) assay showed that from 500 μg/mL, GF, LF and dark roasted freeze-dried (DF) extracts used in the experiments caused a reduction of 50% or more in the viability of the treated prostate tumor cells without significant differences (*p* > 0.05). The extracts that induced the most significant modification in the cell activity were the freeze-dried green and light roasted extracts, with reductions of up to 75% and 73% of the metabolic activity of the tested prostate lineage, respectively (Figure 3A).

Regarding the spray-dried coffee extracts (Figure 3B), it has been shown that when the concentration was increased to 250 μg/mL, all spray-dried extracts started the cellular activity reduction. In small doses, the extracts of medium and dark roasted beans were able to increase the proliferation of prostate tumor cells, while the green and clear extracts promoted reduction in cell growth (*p* < 0.05).

Cells treated with soluble extracts of Robusta coffee reduced the cell viability percentage at a concentration of 250 μg/mL, with a significant difference (*p* < 0.05) compared to the control cells, except in the GS samples. At the concentration of 500 μg/mL, cells showed the highest percentage of inhibition regardless of the type of extract (*p* > 0.05) (Figure 3C). 

Extracts produced at low temperature and freeze-drying were able to reduce the number of viable cells further. Caffeic acid may be associated with reduced risk of prostate cancer, so a diet rich in this acid may have beneficial effects on the reduction of PCa incidence [46]. 5-O-caffeoylquinic acid (5-CQA) is one of the available acids among the phenolic acid compounds that can be found naturally in green coffee extracts and tea. Chlorogenic acid (CGA, 3-CQA) is the most abundant isomer among caffeoylquinic acid isomers (3-, 4-, and 5-CQA), currently known as 5-CQA according to the guidelines of International Union of Pure and Applied Chemistry (IUPAC) [8].

Previous studies evaluate the effects of coffee extracts on the viability on non-cancer cells. The data indicated that coffee extracts were not cytotoxic to AML-12 cells and RAW 264.7 cells [47]. Cell viability reduction showed the roasted coffee extracts in all concentrations tested were noncytotoxic for mouse fibroblasts cells [48]. Additionally, no toxic effects were observed in 3T3-L1 cell line treated with coffee extracts (green and light roasted extracts; see Supplementary Materials).

Table 4 shows the results found in the cell cycle analysis of DU-145 cells treated with soluble extracts of Robusta coffee. We observed different modifications in the phase pattern between cells treated with similar samples, different drying methods and concentrations. In general, in cells treated with lyophilized samples, there was an increase in the number of cells in the G_0_/G_1_ phase and a decrease in G_2_/M, compared to the respective controls. However, in the dark lyophilized sample, for example, no change in the cell cycle pattern was observed. In the spray-dried samples, it was not possible to verify a pattern of modification of the cell cycle. The green spray-dried samples caused a decrease of G_0_/G_1_ and an increase of G_2_/M, different from that found in the freeze-dried samples. Meanwhile, the medium and dark roasted samples showed the same pattern, where there was an increase in the number of cells in the G_0_/G_1_ and G_2_/M reduction phases. In the green spray-dried samples, there was no difference in G_0_/G_1_ compared to untreated cells, but there was an increase in G_2_/M. 

**Figure 4:**
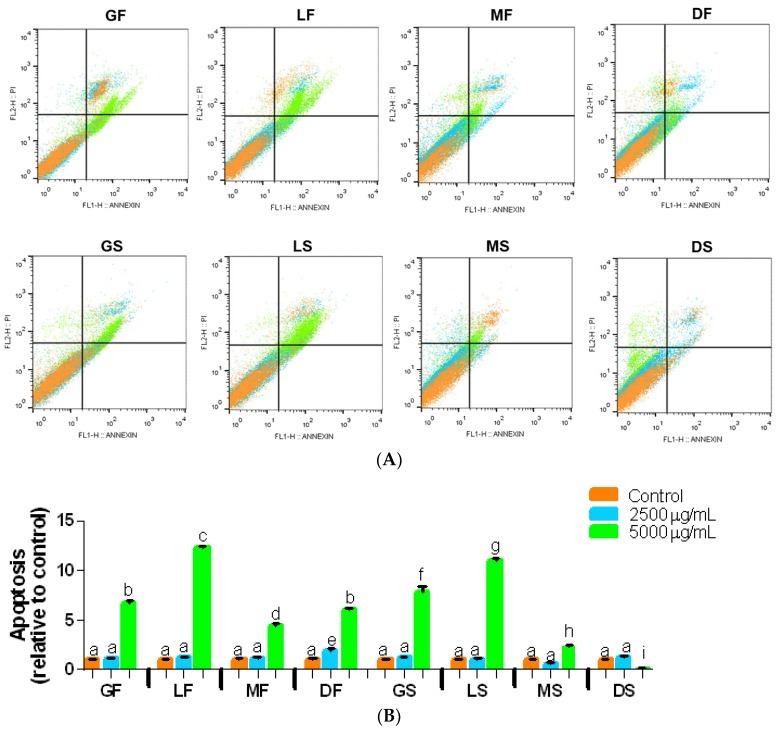
Effect of coffee extracts on rate of apoptosis in DU-145 cells after 24 h after incubation. Flow cytometry analysis of coffee extracts are illustrated in (**A**). (**B**) Relative increase rate of apoptotic cells of DU-145 treated with freeze-dried and spray-dried coffee extracts after 24 h of treatment. Relative comparison between untreated cells (CT) and cells incubated with coffee extracts. Means significant difference with different letters (*p* < 0.05). Abbreviations: GF—green freeze-dried, LF—light roasted freeze-dried, MF—medium roasted freeze-dried, DF—dark roasted freeze-dried, GS—green spray-dried, LS—light roasted spray-dried, MS—medium roasted spray-dried, DS—dark roasted spray-dried.

In the apoptosis assay, cells treated with the soluble coffee extracts showed a population shift to the upper left quadrant of the graph, as can be seen in Figure 4A; there was also displacement to the lower and upper right quadrants, representing the cell populations in early apoptosis and late apoptosis or necrosis. However, the medium and dark extracts had low capacity to modify the cell populations, and these treated with spray-dried extracts had no apoptotic population lower than those observed in control. Apoptosis increased when we used the coffee extract concentration of 5000 µg/mL to treat the cells and the light roasted and green bean extracts were better (Figure 4B).

In Table 5, the number of cells of the control and the samples treated with the coffee extract concentrations of 2500 and 5000 μg/mL for each phase of cell death can be observed. There was an average increase of 11% in apoptosis relative to the control in the cells treated with the light roasted samples at the highest concentration used. According to Murad [16], the intermediate compounds produced by isomerization and hydrolysis of 5-CQA can act positively on the cell cycle modulation and consequently on induction of apoptosis in cells incubated with 5-CQA.

Brazilian coffee for the local market is characterized as having excessive roasting, producing a low-quality drink. The arrival of higher-grade imported coffee in the national market has led to questions about the national standard of roasting. Each country has a characteristic roasting pattern. In Brazil, the roasted coffee is darker not because of consumer preference but due to the need to mask the presence of defects or common alterations in commercial coffee [20].

The literature considers that it is healthy and safe to consume up to four cups per day. Our results support the idea that coffee is a beverage with antioxidant potential, but the extensive processing of the beans influences their content. The degree of roasting is fundamental for the preservation of the healthy characteristics of the beverage, so that the health effects depend not only on the quantity of coffee products consumed but also on the quality of the beans.

There is considerable epidemiological evidence of the preventive effect of coffee consumption on prostate cancer [49]. However, the mechanisms, by which this protection occurs, are still not well understood. Kolberg et al. [50] were the first researchers to suggest mechanistic links between coffee consumption and prostate cancer in an experimental mouse model. In addition, the study showed that coffee could modulate the transcription of genes related to prostate cancer and inflammation. Intervention studies in humans have highlighted that coffee consumption can increase glutathione levels and improve protection against DNA damage, especially when the intake is continuous and with regulated doses. Experimental evidence shows that instant coffee protected lymphoma cells in animals from DNA damage and from gamma radiation in vitro [51,52]. In addition, the consumption of coffee may also be correlated with lower circulating levels of inflammatory markers, including chemokines, cytokines, and the basic fibroblast growth factor (FGF-2), which has been linked to lymphoma pathogenesis [53,54].

It was observed that coffee extracts evaluated presented high percentage of inhibition of oxidation, which can be correlated with substantial contents of these biologically active compounds, and it seems to be a potential source of natural antioxidants for the human diet. However, the positive effects shown in this study may be due to other direct mechanisms that need further research, including inhibition or induction of enzymes, inhibition of receptor activities, and induction and inhibition of gene expression related to apoptosis and cell cycle arrest.

## 3. Materials and Methods

### 3.1. Samples

The green coffee beans from *Coffea canephora* used in this study were purchased from coffee producers in Colatina, Espírito Santo, Brazil. The coffee bags were transported to Rio de Janeiro, stored in the Laboratory for Molecular Diagnosis and Mycology, and processed at the facility of the Embrapa Food Technology in Rio de Janeiro, Brazil.

### 3.2. Bean Processing and Production of Extracts

**Figure 5:**
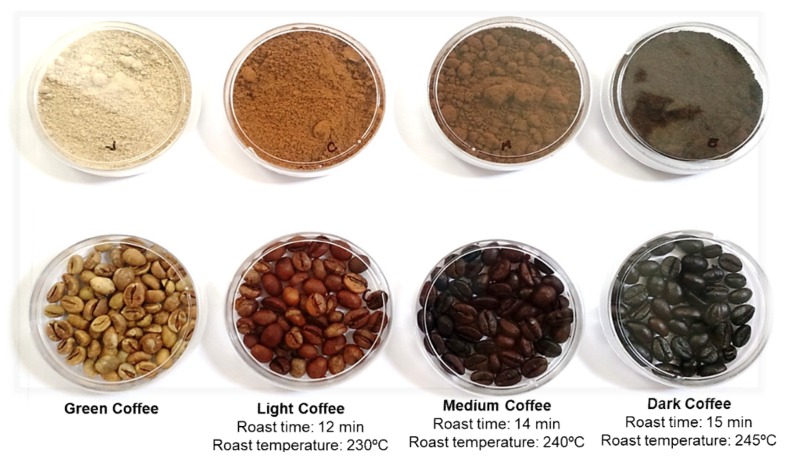
Beans and ground coffee (unroasted or with different levels of roasting).

Green coffee beans were sorted to remove filth and bad beans. Some of the green beans were milled in an analytical grinder (IKA^®^A11 basic, Stalfen, Germany) to produce the green coffee solution and the rest of the beans were roasted in a coffee roaster (Gene Café^®^ CBF-101, Kyungki-Do, Korea). The light roasting was performed at 230 °C for 12 min, medium roasting was conducted at 240 °C for 14 min and the dark roasting was performed at 245 °C for 15 min (Figure 5), according to the Agtron scale [55]. After that, they were milled in a grinder (Cousinart^®^, East Windsor, NJ, USA) and an analytical grinder (IKA^®^A11 basic, Stalfen, Germany). For standardization of the samples, the coffee powders were sieved in an analytical sieve (710 µm). Fifty percentage of the extracts were obtained in hot water (90–95 °C) for 10 min. To optimize the extraction, ultrasound was applied with a sonotrode (Hielscher^®^ UIP1000hdT, Teltow, Germany), followed by immersion in an ice bath (216 W to 60 W; Amplitude = 70% for 10 min). 

The solid particles of the coffee solutions were evaluated on a dry basis and the ^0^Brix value was determined in the initial tests for a yield of the final extracts (data not shown). Then, the solutions were centrifuged (7000 rpm/5 min) and the supernatant of each extract was separated into two parts, one for drying in a freeze drier (dehydration at 60 °C; air velocity of 1 m·s ^−1^ for 32 h) and the other in a spray drier (dehydration at 180 °C; vacuum of −60 mbar). The dehydrated extracts were stored in vacuum-laminated zip-type packages at −80 °C until analysis.

### 3.3. Colorimetric Analysis

The color analyses were performed using a Konica Minolta CM-5 digital colorimeter (NJ, USA). The parameters of L* (lightness), a* (red/green intensity) and b* (yellow/blue intensity) of the CIE-Lab system (*Commission Internationale d’le Ecleraige*) were measured. The analysis was performed in triplicate. The equipment was calibrated following the manufacturer’s instruction manual, using approximately 50 g of a previously homogenized sample [56].

### 3.4. Antioxidant Activity Analyses

#### 3.4.1. Trolox Equivalent Antioxidant Capacity (ABTS/TEAC)

The TEAC^+^ cation was prepared by mixing a TEAC stock solution (7 mM in water) with 2.45 mM of potassium persulfate. This mixture was allowed to stand for 16 h at room temperature until the reaction was completed and the absorbance was stable. The antioxidant capacity assay was carried out by the improved ABTS/TEAC method as described by Rufino et al. [57]. The TEAC solution (2.5 mL) was added to extracts or commercial antioxidant (Trolox) and mixed thoroughly. Absorbance was recorded at 734 nm for 6 min. The results were expressed as µmol Trolox equivalent TE/g dry basis.

#### 3.4.2. DPPH Assay

Aliquots of 0.5 mL of the extracts were mixed with 2.5 mL (2,2 diphenyl-1-picrylhydrazyl (DPPH) methanolic solution (0.06 mM) and allowed to react for 1 h in the dark. Measurements were performed at 515 nm applying a Shimadzu UV-VIS 2700 spectrophotometer (Nakagyo-ku, Kyoto, Japan). The analysis was performed in triplicates; the decline in the DPPH radical absorbance concentration caused by the extracts was compared to a Trolox standard. The results were expressed as µmol TE/g dry basis [58].

#### 3.4.3. Ferric Reducing Ability (FRAP)

The extracts were measured for antioxidant activity by FRAP according to Rufino et al. [50]. Aliquots of 2.7 mL of TPTZ reagent (ferric 2,4,6-tripyridyl-s-triazine) were mixed with 0.5 mL of extract sample. After 30 min at 37 °C, the absorbance was read at 595 nm. The antioxidant capacity was expressed as ferrous sulfate equivalents (µmol FeSO_4_/g dry basis).

#### 3.4.4. ORAC

For ORAC analysis, we used an automatic plate reader (SpectraMax i3x, San Jose, CA, USA) with 96-well plates [59]. Analyses were conducted in a phosphate buffer with pH 7.4 at 37 °C. Peroxyl radical was generated using 2,2′-azobis (2-amidino-propane) dihydrochloride, which was prepared fresh for each run. Fluorescein was used as the substrate. Fluorescence conditions were as follows: excitation at 485 nm and emission at 520 nm. The standard curve was linear between 1 µM and 90 µM Trolox. Results are expressed as mm of TE/g of coffee extract.

### 3.5. Total Phenolic Assay

The total phenolic content of the extracts was determined according to the Folin–Ciocalteu method, as described by Singleton and Rossi [60] with minor modifcations. Aliquots of 0.5 mL of the extracts were added to 2.5 mL of Folin–Ciocalteu reagent and 2.0 mL of 4% sodium carbonate solution, and the mixture was allowed to rest for 2 h in the dark. Measurements were performed at 750 nm in triplicates, applying a Shimadzu UV-VIS 2700 spectrophotometer (Nakagyo-ku, Kyoto, Japan). Gallic acid, in the concentration range of 0–100 mg/mL^−1^, was used to construct a calibration curve. The concentration of total phenolic compounds in the extract was expressed as gallic acid equivalents, which reflect the phenolic content as the amount of gallic acid in mg/100 g dry weight of the samples.

### 3.6. High Performance Liquid Chromatography(HPLC) Analyses

#### 3.6.1. Amino Acids

The method consisted of extracting the amino acids from the sample matrix by acid hydrolysis and subsequent derivatization using the 6-aminoquinolyl-*N*-hydroxysuccinimidyl carbamate reagent. The chromatographic separation was performed with a C_18_ column and gradient with a sodium acetate buffer and acetonitrile. Detection was by fluorescence and quantification by external standardization, except for sulfur amino acids and tryptophan [61,62].

#### 3.6.2. Reducing Sugars

In reducing sugar, the determination method was used to separate the sample by HPLC with normal-phase (amino) column and acetonitrile/water mobile phase, using a refractive index detector and quantification by external standardization [63].

#### 3.6.3. Caffeine

The caffeine content was measured by the HPLC method adapted by Perrone et al. [64], with a Waters Alliance 2695 photodiode array detector (PDA), 2996 chromatograph and Empower^®^ software (Waters, Milford, MA, USA). A Hypersil C_18_ BDS((Base Deactivated Silica) column (5 cm × 4.6 mm and 2.6 μm; Thermo Scientific, Milford, MA, USA) was used. The mobile phase was composed of 10% acetonitrile in a 0.5% (*v*/*v*) acetic acid solution and was also used as a solution for sample extraction: 1 g in 25 mL volumetric flask for 10 min in an ultrasonic bath. The samples were filtered through rapid filter paper and microfiltered into disposable hydrophilic Teflon filter units with a porosity of 0.22 μm. The external caffeine standard was prepared by weighing approximately 30 mg of caffeine in 30 mL, solubilized with the mobile phase. Detection was performed at 272 nm, with a mobile phase flow rate of 0.5 mL/min and an injection volume of 20 μL.

#### 3.6.4. Chlorogenic Acids

The levels of chlorogenic acids and caffeic acid were determined by HPLC, as adapted by Trugo and Macrae [65], using a Waters Alliance 2695 chromatograph, 2996 PDA and Empower^®^ software (Waters, Milford, MA, USA). A Hypersil C_18_ BDS column (5 cm × 4.6 mm and 2.6 μm; Thermo Scientific, Milford, MA, USA) was used. The mobile phase gradient consisted of the initial composition of 5% methanol (phase A) and 95% formic acid (phase B), maintained for 6 min. After 8 min, the composition of the mobile phase reached 80% of phase A and remained there for up to 10 min. After 11 min, the composition reached the plateau of 100% of phase A, and from 12 min to 15 min, the composition returned to the initial conditions. Detection was performed at 320–325 nm, the flow rate of the mobile phase was 1 mL/min and the injection volume was 3 μL. Samples were extracted in an ultrasonic bath for 20 min with 20% acetonitrile in ultrapure water (*v*/*v*). The samples were then centrifuged, microfiltered in disposable hydrophilic Teflon filter units with a porosity of 0.22 μm. A pre-calibrated system with chlorogenic acid and caffeic acid external standards (Sigma-Aldrich, New York, NY, USA) was prepared by weighing about 30 mg of the standard into a solubilized 25 mL of water and pelleted with 0.5% formic acid.

### 3.7. Cell Culture and Treatment Protocol

The cell line was obtained from the Rio de Janeiro Cell Bank, which certified the identity and quality (INMETRO, Rio de Janeiro, RJ, Brazil). The human prostate carcinoma cell line (DU-145) was maintained routinely in RPMI 1640 medium (Sigma, New York, NY, USA), supplemented with 10% fetal bovine serum (FBS) and 1% penicillin-streptomycin (PS) (Sigma, New York, NY, USA), pH 7.4, under 5% CO_2_ atmosphere and 37 °C. Once the cells reached a 80% confluence, they were dissociated with 0.05% trypsin-EDTA(Ethylenediamine tetraacetic acid) and sub-cultured in 25 or 75 cm^2^ plastic flasks at a 25 × 10^4^ cells/cm^2^ density. The culture medium was replaced every 2 days. For each experiment, cells were seeded at a 5 × 10^5^ cells/cm^2^ density in 6-well plates and 2 × 10^4^ cells/cm^2^ densities in 96-well plates for cell cycle and cell proliferation analyses, respectively. After 24 h, the medium was removed and cells were treated with increasing concentrations of extracts of Robusta coffee (25 μg/mL to 5000 μg/mL) dissolved in supplemented RPMI medium. The controls were included in each plate. The cells were then incubated for 24 h.

#### 3.7.1. Cell Viability

Cell viability was monitored by the MTT assay (Amresco, Solon, OH, USA). MTT (3-(4,5-dimethylthiazol-2-yl)-2,5-diphenyltetrazolium bromide) is a pale yellow substrate that is reduced by living cells to yield a blue formazan product. This requires active mitochondria, and even recently, dead cells do not reduce significant amounts of MTT. Exponentially growing cells were adjusted to 2.0 × 104/cm^2^ with RPMI 1640, plated in 96-well plates (Corning, Tewksbury, MA, USA) at 200 µL/well and incubated for 24 h according to the routine procedure. The cells were then incubated with GF, LF, MF or DF, and GS, light spray-dried (LS), medium spray-dried (MS) or dark spray-dried (DS) coffee extracts (25–5000 µg/mL) for 24 h (6 wells for each sample). Each well was also incubated with MTT (10 µL/well; 5 g/mL) for 4 h. After 85 µL/well, the liquid was removed and 50 µL/well of sodium dodecyl sulfate was added to dissolve the solid residue. Finally, the absorbance was measured using a microplate reader (Polaris, CELER^®^, Biotecnologia, Minas Gerais, BH, Brazil) at 570 nm. The cell proliferation inhibition rate (CPIR) was calculated using the following formula:

CPIR = (1 − average value of experimental group/average value of control group) × 100%.

#### 3.7.2. Cell Cycle Analysis

Cells were rinsed briefly with calcium and magnesium-free phosphate-buffered saline (PBS) and detached with trypsin at room temperature. After centrifugation, the cells were washed twice with phosphate-buffered saline; and then resuspended in 500 µL of ice-cold Vindelov solution [66] containing 0.1% Triton X-100, 0.1% citrate buffer, 0.1 mg/mL RNase, and 50 mg/mL propidium iodide (Sigma Chemical Co., St. Louis, MO, USA). After incubation for 15 min, the cell suspension was analyzed for DNA content by flow cytometry using a FACSCalibur flow cytometer (Becton Dickinson, Mountain View, CA, USA). The relative proportions of cells with DNA content indicative of apoptosis (<2n), G_0_/G_1_ diploid (2n), S (2n < phase < 4n), and G_2_/M phase (4n) were obtained and analyzed using the CellQuest WinMDI 2.9 program. 

The percentage of cell population at a particular phase was estimated with FlowJo software. The cell dissociation procedure did not affect fluorescence under the experimental conditions used in this study or in any other studies, of which we are aware. Nuclei of viable cells were gated according to the FL2-width-to-FL2-area relation.

#### 3.7.3. Detection of Apoptosis by Annexin V-FITC

To measure the rate of apoptosis, the cells were subjected to staining with Annexin V conjugated to FITC. The non-adherent cells were collected, and adherent cells were quickly washed with a calcium/magnesium-free buffered saline solution (BSS) and were detached with 0.125% trypsin/EDTA (Sigma Chemical Co., St. Louis, MO, USA) at room temperature. Subsequently, apoptotic and necrotic cells were stained with Annexin V FITC/Propidium Iodide (PI) (BD Pharmingen, Mountain View, CA, USA) according to the manufacturer’s instructions, quantified with a flow cytometer (FACSCalibur, BD Bioscience, Mountain View, CA, USA), and analyzed using two specific programs, Cell Quest and FlowJo.

### 3.8. Statistical Analyses

Results are presented as mean with the corresponding standard deviation of duplicates of two different experiments (*n* = 6). Data were analyzed with the statistical software GraphPad Prism (version 7.0, GraphPad Software, San Diego, CA, USA). One-way analysis of variance (ANOVA) with the Tukey post-hoc test at a confidence level of 95% was used to test cell viability, cell cycle, and apoptosis.

## 4. Conclusions

The physical–chemical data evaluated in the samples from *Coffea canephora* showed that the spray-drying process preserved the antioxidant activity of the extracts better, although it did not had influence on the two bioactive compounds evaluated specifically (chlorogenic acid and caffeic acid). The drying process, however, depends on the binomial time and temperature. The higher preservation of antioxidant activity in the spray-drying processing can be associated with a shorter exposure in a more inert environment (vacuum system) that would better protect other bioactive components, despite the higher temperature. 

Finally, the extracts from green and light roasted beans exhibited a strong bioactive capacity. In addition, coffee extracts promoted a decrease in cell viability, modulated cell cycle and induced apoptosis in DU-145 prostate cancer cells. Our study has far-reaching health relevance as coffee could be projected as chemopreventive and chemotherapeutic medicine which, in addition to providing nutrition, can contribute to prevent cancer development and progression.

## Figures and Tables

**Table 1:** Colorimetric coordinates (L* a* b*) of the dry extracts of Robusta coffee.

	GF	LF	MF	DF	GS	LS	MS	DS
Coordinate								
L*	58.9 ± 0.1 ^a^	28.6 ± 0.0 ^b^	27.2 ± 0.0 ^c^	27.4 ± 0.0 ^d^	73.5 ± 0.0 ^e^	54.2 ± 0.0 ^f^	51.1 ± 0.0 ^g^	57.5 ± 0.0 ^h^
a*	2.6 ± 0.0 ^a^	9.1 ± 0.0 ^b^	7.5 ± 0.0 ^c^	7.4 ± 0.0 ^d^	0.4 ± 0.0 ^e^	8.9 ± 0.0 ^f^	8.2 ± 0.0 ^g^	8.7 ± 0.0 ^h^
b*	17.8 ± 0.0 ^a^	11.0 ± 0.0 ^b^	9.9 ± 0.0 ^c^	9.8 ± 0.0 ^d^	15.9 ± 0.0 ^e^	20.9 ± 0.0 ^f^	19.1 ± 0.0 ^g^	18.8 ± 0.0 ^h^

(a–h) Different letters in the same attribute mean that samples are different (*p* < 0.05) and same letters indicate the samples are the same (*p* > 0.05). Abbreviations: GF—green freeze-dried, LF—light roasted freeze-dried, MF—medium roasted freeze-dried, DF—dark roasted freeze-dried, GS—green spray-dried, LS—light roasted spray-dried, MS—medium roasted spray-dried, DS—dark roasted spray-dried.

**Table 2:** Results of sugars and some amino acids for freeze-dried and spray-dried coffee extracts.

	GF	LF	MF	DF	GS	LS	MS	DS
**Sugars (g/100 g)**								
Sucrose	11.6 ± 0.1 ^a^	ND	ND	ND	11.8 ± 0.2 ^a^	ND	ND	ND
Fructose	1.4 ± 0.1 ^a^	ND	ND	ND	1.3 ± 0.0 ^a^	ND	ND	ND
**Amino Acids (g/100 g)**								
Asparagine	1.5 ± 0.1 ^a^	0.6 ± 0.0 ^b^	0.6 ± 0.0 ^b^	0.4 ± 0.1 ^c^	1.5 ± 0.1 ^a^	0.4 ± 0.1 ^b,c^	0.6 ± 0.0 ^b,c^	0.4 ± 0.1 ^c^
Glutamine	3.4 ± 0.2 ^a^	2.2 ± 0.0 ^b^	2.8 ± 0.1 ^a,b^	2.5 ± 0.4 ^a,b^	3.4 ± 0.2 ^a^	1.5 ± 0.2 ^c^	2.8 ± 0.3 ^d^	2.6 ± 0.4 ^e^
Histidine	0.3 ± 0.0 ^a^	0.2 ± 0.0 ^b^	0.2 ± 0.0 ^b^	0.1 ± 0.0 ^c^	0.4 ± 0.1 ^a^	0.1 ± 0.0 ^b,c^	0.2 ± 0.01 ^b,c^	0.1 ± 0.0 ^c^
Arginine	1.0 ± 0.0 ^a^	0.2 ± 0.0 ^b^	0.1 ± 0.0 ^b^	0.1 ± 0.0 ^b^	1.0 ± 0.0 ^a^	0.1 ± 0.0 ^b^	0.1 ± 0.0 ^b^	0.1 ± 0.0 ^b^
Proline	1.1 ± 0.0 ^a^	0.6 ± 0.0 ^b^	0.6 ± 0.0 ^b^	0.5 ± 0.1 ^b^	1.2 ± 0.0 ^a^	0.4 ± 0.0 ^b,c^	0.6 ± 0.7 ^b^	0.5 ± 0.1 ^b^
Leucine	1.2 ± 0.1 ^a^	0.6 ± 0.0 ^b^	0.7 ± 0.0 ^b^	0.6 ± 0.1 ^b^	1.3 ± 0.0 ^a^	0.4 ± 0.1 ^b,c^	0.8 ± 0.1 ^b^	0.6 ± 0.1 ^b^
Phenylalanine	0.8 ± 0.0 ^a^	0.4 ± 0.0 ^b^	0.4 ± 0.0 ^b^	0.3 ± 0.0 ^b^	0.9 ± 0.0 ^a^	0.3 ± 0.4 ^b^	0.4 ± 0.1 ^b^	0.3 ± 0.0 ^b^

ND: not detected. (a–e) Different letters in the same attribute mean that samples are different (*p* < 0.05) and same letters indicate the samples are the same (*p* > 0.05). Abbreviations: GF—green freeze-dried, LF—light roasted freeze-dried, MF—medium roasted freeze-dried, DF—dark roasted freeze-dried, GS—green roasted spray-dried, LS—light roasted spray-dried, MS—medium roasted spray-dried, DS—dark roasted spray-dried.

**Table 3:** Results of caffeine and total phenolic compounds for freeze-dried and spray-dried extracts.

	GF	LF	MF	DF	GS	LS	MS	DS
Caffeine (g/100 g)	0.3 ± 0.0 ^a^	0.2 ± 0.1 ^b^	0.2 ± 0.0 ^b^	0.1 ± 0.0 ^c^	0.4 ± 0.1 ^a^	0.2 ± 0.0 ^b,c^	0.2 ± 0.0 ^b,c^	0.1 ± 0.0 ^c^
Total Phenolic compounds (mg of gallic ac./100 g)	3051.1 ± 33.7 ^a^	3792.0 ± 13.4 ^b^	2177.2 ± 70.8 ^c^	2198.7 ± 71.0 ^c^	2816.6 ± 19.2 ^a^	3046.4 ± 64.0 ^a^	1889.0 ± 24.6 ^d^	1437.1 ± 17.5 ^e^

(a–e) Different letters in the same attribute mean that samples are different and same letters indicate the samples are the same. Abbreviations: GF—green freeze-dried, LF—light roasted freeze-dried, MF—medium roasted freeze-dried, DF—dark roasted freeze-dried, GS— green freeze-dried, LS—light roasted spray-dried, MS—medium roasted spray-dried, DS—dark roasted spray-dried.

**Table 4:** Effects of freeze-dried and spray-dried coffee extracts (2500 and 5000 µg/mL) on cell cycle progression in human prostate cancer cell line after 24 h of treatment.

Sample	Cell Cycle Phase	Control	2500 µg/mL	5000 µg/mL
**GF**	G_0_/G_1_	50.8 ± 3.1 ^a^	64.8 ± 2.1 ^b^	75.6 ± 0.5 ^c^
	S	6.1 ± 2.7 ^a^	6.6 ± 1.0 ^a^	8.4 ± 2.9 ^a^
	G_2_/M	44.3 ± 1.0 ^a^	30.2 ± 3.1 ^b^	13.5 ± 2.8 ^c^
**LF**	G_0_/G_1_	53.9 ± 3.3 ^a^	54.1 ± 2.9 ^a^	63.4 ± 4.6 ^b^
	S	8.8 ± 1.4 ^a^	9.8 ± 2.1 ^a^	4.6 ± 1.0 ^b^
	G_2_/M	39.8 ± 3.4 ^a^	36.4 ± 4.2 ^a,b^	30.1 ± 1.3 ^b^
**MF**	G_0_/G_1_	59.3 ± 2.0 ^a^	71.8 ± 1.4 ^b^	85.2 ± 3.2 ^c^
	S	5.6 ± 1.3 ^a^	4.7 ± 1.0 ^a^	2.9 ± 0.5 ^a^
	G_2_/M	34.2 ± 1.0 ^a^	22.4 ± 2.1 ^b^	11.0 ± 3.1 ^c^
**DF**	G_0_/G_1_	51.1 ± 0.1 ^a^	51.0 ± 8.0 ^a^	39.7 ± 3.1 ^a,b^
	S	6.2 ± 0.1 ^a^	5.6 ± 0.4 ^a^	11.0 ± 0.4 ^b^
	G_2_/M	41.8 ± 0.1 ^a^	42.6 ± 8.0 ^a^	48.3 ± 2.6 ^a^
**GS**	G_0_/G_1_	50.0 ± 3.3 ^a^	45.1 ± 4.2 ^a^	34.4 ± 4.6 ^b^
	S	7.56 ± 1.3 ^a^	9.7 ± 2.2 ^b^	8.3 ± 3.2 ^c^
	G_2_/M	44.6 ± 3.0 ^a^	46.0 ± 3.4 ^a^	58.9 ± 3.0 ^b^
**LS**	G_0_/G_1_	61.5 ± 11.0 ^a^	65.1 ± 1.1 ^a^	52.1 ± 3.7 ^a^
	S	12.4 ± 4.9 ^a^	8.0 ± 1.2 ^a^	10.5 ± 0.6 ^a^
	G_2_/M	25.0 ± 7.0 ^a^	25.8 ± 2.4 ^a^	36.4 ± 4.3 ^a,b^
**MS**	G_0_/G_1_	54.3 ± 0.2 ^a^	22.3 ± 3.7 ^b^	70.4 ± 7.1 ^c^
	S	7.0 ± 0.3 ^a^	3.8 ± 0.4 ^a^	11.5 ± 1.9 ^b^
	G_2_/M	37.5 ± 0.4 ^a^	72.6 ± 2.6 ^b^	18.9 ± 3.1 ^c^
**DS**	G_0_/G_1_	56.2 ± 2.8 ^a^	65.1 ± 1.0 ^b^	83.0 ± 0.7 ^c^
	S	4.0 ± 0.2 ^a^	7.2 ± 1.7 ^b^	3.7 ± 0.2 ^a^
	G_2_/M	38.3 ± 2.9 ^a^	27.0 ± 1.2 ^b^	12.9 ± 0.5 ^c^

Results are expressed as the percentage of total cells. The data represent mean ± standard deviation values of triplicate experiments. Tukey test: (a–c) different letters in the same phase and extract means that samples are different (*p* < 0.05) and same letters indicate samples are the same (*p* > 0.05). Abbreviations: GF—green freeze-dried, LF—light roasted freeze-dried, MF—medium roasted freeze-dried, DF—dark roasted freeze-dried, GS—green spray-dried, LS—light roasted spray-dried, MS—medium roasted spray-dried, DS—dark roasted spray-dried.

**Table 5:** Effect of freeze-dried and spray-dried coffee extracts (2500 and 5000 µg/mL) on programmed cell death in a human prostate cancer cell line after 24 h of treatment.

Sample	Phases of Cell Death Process	CT	2500 µg/mL	5000 µg/mL	Sample	Phases of Cell Death Process	CT	2500 µg/mL	5000 µg/mL
**GF**	**Viable cells**	86.4 ± 0.6 ^a^	85.3 ± 0.9 ^a^	20.3 ± 16.1 ^b^	**GS**	**Viable cells**	90.0 ± 1.8 ^a^	87.2 ± 0.6 ^a^	15.7 ± 0.8 ^a^
	**Early apoptosis**	1.9 ± 0.3 ^a^	1.8 ± 0.4 ^a^	55.3 ± 5.0 ^b^		**Early apoptosis**	5.7 ± 0.5 ^a^	5.9 ± 1. 5 ^a^	30.8 ± 10.8 ^a^
	**Late apoptosis/Necrosis**	11.2 ± 1.0 ^a^	12.4 ± 1.4 ^a^	23.6 ± 11.5 ^a^		**Late apoptosis/Necrosis**	3.9 ± 1.4 ^a^	6.7 ± 2.2 ^a^	52.0 ± 10.2 ^b^
	**Non-apoptotic cells**	0.5 ± 0.1 ^a^	0.4 ± 0.1 ^a^	0.8 ± 0.3 ^a^		**Non-apoptotic cells**	0.3 ± 0.1 ^a^	0.2 ± 0.1 ^a^	1.4 ± 0.3 ^b^
**LF**	**Viable cells**	92.3 ± 0.8 ^a^	92.9 ± 0.6 ^a^	14.8 ± 4.7 ^b^	**LS**	**Viable cells**	91.9 ± 2.2 ^a^	92.2 ± 0.4 ^a^	9.7 ± 1.4 ^b^
	**Early apoptosis**	1.4 ± 0.3 ^a^	4.3 ± 0.1 ^a^	39.4 ± 1.4 ^b^		**Early apoptosis**	3.3 ± 1.7 ^a^	4.2 ± 0.5 ^a^	26.9 ± 4.4 ^b^
	**Late apoptosis/Necrosis**	5.3 ± 1.0 ^a^	2.9 ± 0.7 ^a^	44.4 ± 3.6 ^b^		**Late apoptosis/Necrosis**	4.6 ± 0.5 ^a^	3.4 ± 0.1 ^a^	62.2 ± 3.2 ^b^
	**Non-apoptotic cells**	0.9 ± 0.5 ^a^	0.1 ± 0.0 ^a^	1.4 ± 0.4 ^a^		**Non-apoptotic cells**	0.3 ± 0.0 ^a^	0.1 ± 0.0 ^a^	1.0 ± 0.1 ^a^
**MF**	**Viable cells**	95.2 ± 1.0 ^a^	90.4 ± 1.2 ^a^	69.3 ± 5.6 ^b^	**MS**	**Viable cells**	89.5 ± 1.1 ^a^	92.1 ± 0.2 ^b^	72.7 ± 1.7 ^c^
	**Early apoptosis**	0.6 ± 0.1 ^a^	3.2 ± 0.5 ^a^	18.4 ± 5.3 ^b^		**Early apoptosis**	2.2 ± 0.2 ^a^	3.2 ± 0.4 ^a^	14.5 ± 0.4 ^b^
	**Late apoptosis/Necrosis**	3.1 ± 1.0 ^a^	5.3 ± 1.1 ^b^	8.6 ± 1.2 ^c^		**Late apoptosis/Necrosis**	7.3 ± 1.0 ^a^	2.0 ± 0.2 ^b^	10.6 ± 0.2 ^c^
	**Non-apoptotic cells**	1.2 ± 0.5 ^a^	1.0 ± 0.3 ^a^	3.6 ± 0.8 ^b^		**Non-apoptotic cells**	1.0 ± 0.1 ^a^	2.6 ± 0.0 ^b^	2.2 ± 0.4 ^b^
**DF**	**Viable cells**	90.4 ± 4.4 ^a^	89.6 ± 0.4 ^a^	74.3 ± 3.5 ^b^	**DS**	**Viable cells**	93.6 ± 0.4 ^a^	90.5 ± 0.9 ^a^	96.6 ± 0.1 ^a^
	**Early apoptosis**	1.6 ± 1.0 ^a^	3.7 ± 0.2 ^a^	18.4 ± 2.2 ^b^		**Early apoptosis**	2.7 ± 0.2 ^a^	3.9 ± 0.5 ^b^	0.1 ± 0.0 ^c^
	**Late apoptosis/Necrosis**	6.7 ± 4.4 ^a^	5.6 ± 0.2 ^a^	4.5 ± 1.2 ^a^		**Late apoptosis/Necrosis**	3.1 ± 0.1 ^a^	4.2 ± 0.8 ^a^	0.1 ± 0.0 ^b^
	**Non-apoptotic cells**	1.3 ± 0.9 ^a^	1.1 ± 0.0 ^a^	2.6 ± 0.2 ^b^		**Non-apoptotic cells**	0.7 ± 0.0 ^a^	1.4 ± 0.1 ^b^	3.3 ± 0.1 ^c^

Results are expressed as the percentage of total cells. The data represent mean ± standard deviation values of triplicate experiments. Tukey test: (a–c) different letters in the same phase of death process and extract means that samples are different (*p* < 0.05) and same letters indicate samples are the same (*p* > 0.05). Abbreviations: GF—green freeze-dried, LF—light roasted freeze-dried, MF—medium roasted freeze-dried, DF—dark roasted freeze-dried, GS—green spray-dried, LS—light roasted spray-dried, MS—medium roasted spray-dried, DS—dark roasted spray-dried. Viable cells (Annexin V−/PI−), Early apoptosis (Annexin V+/PI−), Late apoptosis/Necrosis (Annexin V+/PI+), Non-apoptotic cells (Annexin V−/PI+). PI—propidium iodide.

## Supplementary Materials

Supplementary materials can be found at http://www.mdpi.com/1422-0067/19/11/3407/s1.

## Abbreviations

ABTS/TEAC (Trolox equivalent antioxidant capacity); CGA (chlorogenic acids); CPIR (cell proliferation inhibition rate); DF (dark roasted freeze-dried extract); DPPH (2,2-diphenyl-1-picrylhydrazyl); DS (dark roasted spray-dried extract); DU-145 (human prostate carcinoma cell line); FBS (fetal bovine serum); FRAP (ferric reducing ability); GF (green freeze-dried extract); GS (green spray-dried extract); HPLC (high-performance liquid chromatography); L* (lightness); LF (light roasted freeze-dried extract); LS (light roasted spray-dried extract); MF (medium roasted freeze-dried extract); MS (medium roasted spray-dried extract); MTT (3-(4,5-dimethylthiazol-2-yl)-2,5-diphenyltetrazolium bromide); OH (hydroxyl); ORAC (oxygen radical absorbance capacity); PBS (phosphate-buffered saline); PCa (prostate cancer); PI (propidium iodide); PS (penicillin-streptomycin).
