# Supplementary material for: Effect of Roasting Levels and Drying Process of Coffea canephora on the Quality of Bioactive Compounds and Cytotoxicity
Source: Int J Mol Sci. 2018 Oct 31;19(11):3407. doi: 10.3390/ijms19113407 (PMC6274859; doi:10.3390/ijms19113407)
Supplement: Supplementary file 1 [file ijms-19-03407-s001.pdf]

International Journal of *Molecular Sciences*

MDPI

Supplementary material

S1. Complete amino acids results for coffee extracts of freeze-dried and spray-dried process.

Amino Acids (g/100g)	GF	LF	MF	DF	GS	LS	MS	DS
Asparagine	1.48 ± 0.11^{a}	0.63 ± 0.01^{b}	0.59 ± 0.02^{b}	0.36 ± 0.06^{c}	1.48 ± 0.10^{a}	0.43 ± 0.05^{b,c}	0.57 ± 0.04^{b,c}	0.37 ± 0.06^{c}
Serine	0.73 ± 0.04^{a}	0.25 ± 0.01^{b}	0.08 ± 0.01^{c}	0.05 ± 0.01^{c}	0.74 ± 0.01^{a}	0.18 ± 0.02^{d}	0.07 ± 0.00^{c}	0.04 ± 0.00^{c}
Glutamine	3.42 ± 0.23^{a}	2.23 ± 0.01^{b}	2.79 ± 0.10^{a,b}	2.51 ± 0.40^{a,b}	3.41 ± 0.20^{a}	1.49 ± 0.21^{c}	2.79 ± 0.26^{d}	2.63 ± 0.42^{e}
Glycine	0.85 ± 0.05^{a}	0.47 ± 0.01^{b}	0.48 ± 0.01^{b}	0.35 ± 0.06^{b}	0.87 ± 0.03^{a}	0.34 ± 0.04^{b}	0.48 ± 0.06^{b}	0.39 ± 0.04^{b}
Histidine	0.35 ± 0.00^{a}	0.18 ± 0.01^{b}	0.16 ± 0.00^{b}	0.12 ± 0.00^{c}	0.37 ± 0.10^{a}	0.15 ± 0.01^{b,c}	0.15 ± 0.01^{b,c}	0.13 ± 0.01^{c}
Arginine	1.01 ± 0.04^{a}	0.17 ± 0.01^{b}	0.14 ± 0.01^{b}	0.11 ± 0.00^{b}	0.99 ± 0.02^{a}	0.12 ± 0.00^{b}	0.12 ± 0.01^{b}	0.10 ± 0.01^{b}
Threonine	0.53 ± 0.04^{a}	0.23 ± 0.01^{b}	0.14 ± 0.01^{c}	0.06 ± 0.00^{d}	0.52 ± 0.00^{a}	0.17 ± 0.01^{b,c}	0.13 ± 0.01^{c}	0.06 ± 0.00^{d}
Alanine	0.61 ± 0.04^{a}	0.32 ± 0.01^{b}	0.37 ± 0.01^{b}	0.29 ± 0.04^{b}	0.62 ± 0.03^{a}	0.19 ± 0.04^{b,c}	0.23 ± 0.16^{b}	0.30 ± 0.04^{b}
Proline	1.10 ± 0.05^{a}	0.65 ± 0.01^{b}	0.64 ± 0.01^{b}	0.49 ± 0.08^{b}	1.18 ± 0.03^{a}	0.43 ± 0.01^{b,c}	0.59 ± 0.71^{b}	0.50 ± 0.07^{b}
Tyrosine	0.61 ± 0.06^{a}	0.40 ± 0.01^{b}	0.37 ± 0.01^{b}	0.29 ± 0.01^{b,c}	0.65 ± 0.02^{a}	0.29 ± 0.03^{a}	0.35 ± 0.01^{b}	0.29 ± 0.01^{b,c}
Valine	0.76 ± 0.04^{a}	0.33 ± 0.01^{b}	0.41 ± 0.01^{b}	0.32 ± 0.04^{b}	0.78 ± 0.01^{a}	0.24 ± 0.03^{b,c}	0.40 ± 0.04^{b}	0.35 ± 0.05^{b}
Lysine	0.85 ± 0.05^{a}	0.09 ± 0.00^{b}	0.09 ± 0.00^{b}	0.07 ± 0.00^{b}	0.79 ± 0.06^{a}	0.08 ± 0.00^{b}	0.08 ± 0.00^{b}	0.07 ± 0.00^{b}
Isoleucine	0.55 ± 0.03^{a}	0.28 ± 0.01^{b}	0.32 ± 0.01^{b}	0.24 ± 0.03^{b}	0.57 ± 0.01^{a}	0.21 ± 0.02^{b,c}	0.32 ± 0.04^{b}	0.26 ± 0.03^{b}
Leucine	1.24 ± 0.07^{a}	0.61 ± 0.01^{b}	0.75 ± 0.01^{b}	0.57 ± 0.08^{b}	1.28 ± 0.03^{a}	0.44 ± 0.06^{b,c}	0.76 ± 0.11^{b}	0.62 ± 0.08^{b}
Phenylalanine	0.82 ± 0.04^{a}	0.39 ± 0.01^{b}	0.42 ± 0.01^{b}	0.30 ± 0.03^{b}	0.89 ± 0.02^{a}	0.31 ± 0.44^{b}	0.42 ± 0.05^{b}	0.34 ± 0.03^{b}

* Different letters in the same attribute mean that samples are different and same letters indicate the samples are the same.

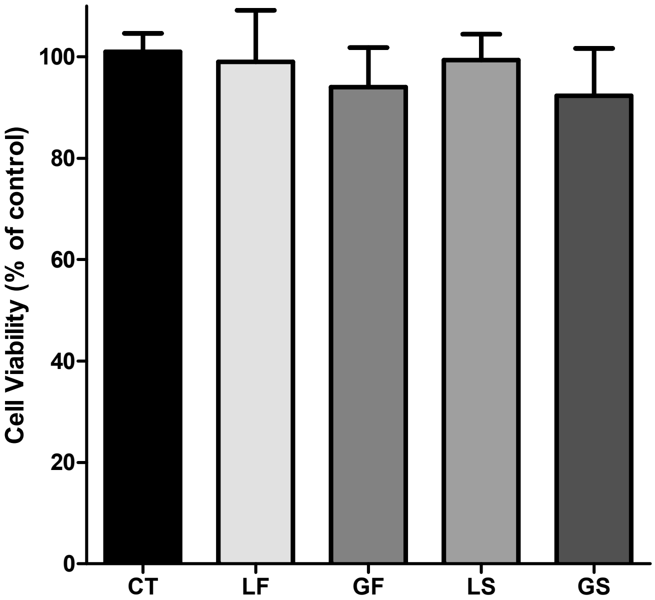

S2. Percentage inhibition in comparison with control of viability of murine fibroblast cell line (3T3 cells) by MTT method, after 24 hours of treatment with freeze-dried (A) and spray-dried (B) aqueous coffee extracts at concentrations of 2500 to 5000 µg/mL. Abbreviations: GF – green freeze-dried, LF – light roasted freeze-dried, GS – green spray-dried, LS – light roasted spray-dried.
